# The association between physical multimorbidity and fall-related injury among adults aged ≥ 50 years from low- and middle-income countries

**DOI:** 10.1007/s10433-025-00848-y

**Published:** 2025-03-20

**Authors:** Lee Smith, Guillermo F. López Sánchez, Jae Il Shin, Hans Oh, Karel Kostev, Mark A. Tully, Yvonne Barnett, Laurie T. Butler, Nicola Veronese, Pinar Soysal, Louis Jacob, Ai Koyanagi

**Affiliations:** 1 Centre for Health Performance and Wellbeing, Anglia Ruskin University, Cambridge, UK; 2 Division of Preventive Medicine and Public Health, Department of Public Health Sciences, School of Medicine, University of Murcia, Murcia, Spain; 3 Department of Pediatrics, Yonsei University College of Medicine, Seoul, Republic of Korea; 4 Institute of Convergence Science, Severance Underwood Meta-Research Center, Yonsei University, Seoul, Republic of Korea; 5 Suzanne Dworak Peck School of Social Work, University of Southern California, Los Angeles, CA USA; 6 University Clinic of Marburg, Marburg, Germany; 7 School of Medicine, Ulster University, Londonderry, Northern Ireland UK; 8 Saint Camillus International University of Health Sciences, Faculty of Medicine, Rome, Italy; 9 Department of Geriatric Medicine, Faculty of Medicine, Bezmialem Vakif University, Istanbul, Turkey; 10 Research and Development Unit, Parc Sanitari Sant Joan de Déu, Dr. Antoni Pujadas, Sant Boi de Llobregat, Barcelona, Spain; 11 Assistance Publique - Hôpitaux de Paris (AP-HP) / Université Paris Cité, Lariboisière - Fernand Widal Hospital, Department of Physical Medicine and Rehabilitation, Paris, France; 12 Inserm / Université Paris Cité, UMR 1153, CRESS, Epidemiology of Ageing and Neurodegenerative Diseases (EpiAgeing), Paris, France

**Keywords:** Low-and middle-income countries, Falls, Multimorbidity, Chronic disease, Epidemiology

## Abstract

Studies from high-income countries have shown that multimorbidity is associated with increased fall risk among older adults. However, studies specifically on this topic from low- and middle-income counties (LMICs) are lacking. Thus, we aimed to assess this association among adults aged ≥ 50 years from six LMICs.

Cross-sectional, community-based data from the Study on Global Ageing and Adult Health (SAGE) were analyzed. Eleven chronic physical conditions were assessed. The presence of past 12-month fall-related injury was ascertained through self-reported information. Multivariable logistic regression and mediation analysis was conducted to assess the association between multimorbidity and fall-related injury.

Data on 34,129 adults aged ≥ 50 years [mean (SD) age 62.4 (16.0) years; males 48.0%] were analyzed. Overall, compared to having no chronic conditions, having 2, 3, and ≥ 4 chronic conditions were significantly associated with 1.67 (95%CI = 1.21–2.30), 2.64 (95%CI = 1.89–3.68), and 3.67 (95%CI = 2.42–5.57) times higher odds for fall-related injury. The association between multimorbidity (i.e., ≥ 2 chronic conditions) and fall-related injury was mainly explained by pain/discomfort (mediated% 39.7%), mobility (34.1%), sleep/energy (24.2%), and cognition (13.0%).

Older adults with multimorbidity in LMICs are at increased odds for fall-related injury. Targeting the identified potential mediators among those with multimorbidity may reduce fall risk in this population.

**Supplementary Information:**

The online version contains supplementary material available at 10.1007/s10433-025-00848-y.

## Introduction

A fall is defined as an event which results in a person coming to rest inadvertently on the ground or floor or other lower level (World Health Organization 2021a). Approximately 37.3 million falls severe enough to require medical attention occur each year. Furthermore, each year, an estimated 684 000 individuals die from falls globally, of which over 80% are in low- and middle-income countries (LMICs) (World Health Organization 2021a). Worldwide, falls are responsible for over 38 million disability-adjusted life years lost each year (World Health Organization 2021a). Literature suggests that older adults are at the highest risk of falling owing to balance problems, muscle weakness, vision loss, and long-term chronic conditions (National Health Service 2021; Kushkestani et al. 2022). Fall-related injury is also a substantial economic burden on society. For example, among older adults aged ≥ 65 years, the average health system cost per fall injury in Finland and Australia are US$ 3611 and US$ 1049, respectively (World Health Organization 2021a).

It is clear that interventions are needed to prevent falls in older adults. To aid in doing so, correlates or risk factors of falls among the interested population need to be identified in order to target those at highest risk. Previously reported risk factors for falls in older adults include for example, difficulties in instrumental activities of daily living (Bloch et al. 2010), mental health complications (e.g., depression) (Iaboni and Flint 2013), fear of falling (Young and Williams 2015) and some medications (Nguyen and Watanabe 2019). However, one potential understudied correlate of falls in older adults is that of physical multimorbidity. Multimorbidity is commonly understood to be the coexistence of multiple health conditions in an individual (Johnston et al. 2019). Physical multimorbidity is feasibly related to an increased risk of falls through factors such as a decrease in functional ability, frailty, and polypharmacy (Afrin et al. 2016; Vetrano et al. 2019; Esparza Montero 2021). Moreover, physical multimorbidity may lead to falls via mental health complications, sleep problems, pain, and cognitive decline (Arokiasamy et al. 2015; Scherer et al. 2016; Wei et al. 2020).

Although mainly from high-income countries, the few previous studies on the association between multimorbidity and falls have shown that those with multimorbidity are at increased risk of falls. For example, in a cross-sectional study of 16,357 community-dwelling Canadian adults aged ≥ 65 years, it was found that risk of falling increased as a function of the number of chronic conditions (Sibley et al. 2014). In another study of 12,669 community-dwelling older adults receiving Medicare in the United States, it was shown that the likelihood of having had a fall in the previous year (relative to no falls) was associated with increasing number of comorbidities (Shumway-Cook et al. 2009). Furthermore, in a study of 872 older persons from Finland, it was observed that recurrent fallers lived with a higher number of diseases (median 4, interquartile range, IQR = 2.0–5.0) compared to non-fallers (median 2, IQR = 1.0–3.0) (Immonen et al. 2020). Other studies from high income settings have found similar results (Clerencia-Sierra et al. 2015; Teixeira et al. 2019).

However, there is only one single country study from a LMIC that has focused specifically on the association of multimorbidity and falls (Barik et al. 2022). This study was carried out in a sample of 28,567 participants aged ≥ 60 years from Longitudinal Ageing Study in India it was observed that multimorbidity [AOR: 1.29 (1.14–1.46)] was significantly associated with falls (Barik et al. 2022). This is an important research gap as disease profiles may differ between high-income countries and LMICs, while treatment options for many chronic diseases may be limited in LMICs. In addition, previous studies on this topic only focused on a single country, and multi-country studies are lacking. Multi-country studies are of particular importance given that they can provide information on whether an association is context-specific or can be generalized across regions. Finally, none of the previous studies have quantified the extent to which potential mediators may explain the association between multimorbidity and falls. Identifying such mediators can provide insights into the mechanisms that link multimorbidity and falls, and therefore help to inform targeted interventions.

Given this background, the aim of the present study is to examine the association between multimorbidity and fall-related injury in a sample of 34,129 adults aged ≥ 50 years from six LMICs. A further aim was to investigate to what extent depression, weak handgrip strength, sleep/energy, pain/discomfort, cognition, and mobility mediate this association.

## Methods

### Sample and study design

Data from the Study on Global Ageing and Adult Health (SAGE) Wave 1 were analyzed (http://www.who.int/healthinfo/sage/en/). This survey was undertaken in China, Ghana, India, Mexico, Russia, and South Africa between 2007 and 2010. Based on the World Bank classification at the time of the survey, all these countries were LMICs. Details of the survey methodology have been published elsewhere (Kowal et al. 2012). Briefly, nationally representative samples were obtained using a multistage clustered sampling design method. The sample consisted of adults aged ≥ 18 years with oversampling of participants aged ≥ 50 years. Standard translation procedures were conducted to ensure comparability between countries, and trained interviewers conducted face-to-face interviews using a standard questionnaire. The survey response rates were: China 93%; Ghana 81%; India 68%; Mexico 53%; Russia 83%; and South Africa 75%. Sampling weights were calculated to adjust for the population structure as reported by the United Nations Statistical Division. Ethical approval was obtained from the WHO Ethical Review Committee and local ethics research review boards, and written informed consent was obtained from all participants.

### Fall-related injury

The variable on fall-related injury included in the SAGE was derived from questions of the WHO guidelines on injuries (Williams et al. 2015). First, the participant was asked “In the past 12 months, have you had any other event (other than a road traffic accident) where you suffered from bodily injury?” Those who answered affirmatively were prompted to the next question “What was the cause of the injury?” If there were multiple injuries, the respondent was instructed to refer to the most recent injury. If the respondent answered “Fall”, then he or she was considered to have had a fall-related injury in the past year.

### Chronic conditions and multimorbidity

We included all 11 chronic physical conditions (angina, arthritis, asthma, chronic back pain, chronic lung disease, diabetes, edentulism, hearing problems, hypertension, stroke, visual impairment) for which data were available in the SAGE. Chronic back pain was defined as having had back pain every day during the last 30 days. Respondents who answered affirmatively to the question “Have you lost all of your natural teeth?” were considered to have edentulism. The participant was considered to have hearing problems if the interviewer observed this condition during the survey. Hypertension was defined as having at least one of the following: systolic blood pressure ≥ 140 mmHg; diastolic blood pressure ≥ 90 mmHg; or self-reported diagnosis. Visual impairment was defined as having severe/extreme difficulty in seeing and recognizing a person that the participant knows across the road (Freeman et al. 2013). Diabetes and stroke were solely based on lifetime self-reported diagnosis. For other conditions, the participant was considered to have the condition in the presence of either one of the following: self-reported diagnosis; or symptom-based diagnosis based on algorithms. We used these algorithms, which have been used in previous studies using the same dataset, to detect undiagnosed cases (Garin et al. 2016; Arokiasamy et al. 2017). Specifically, the validated Rose questionnaire was used for angina (Rose 1962), and other previously validated symptom-based algorithms were used for arthritis, asthma, and chronic lung disease (Arokiasamy et al. 2017). Further details on the definition of chronic physical conditions can be found in Table S1 (supplementary material). The total number of chronic physical conditions was calculated and categorized as 0, 1, 2, 3, and ≥ 4. Multimorbidity was defined as ≥ 2 chronic physical conditions, in line with previously used definitions (Garin et al. 2016).

### Mediators

The mediators in this study (i.e., depression, weak handgrip strength, sleep/energy, pain/discomfort, cognition, mobility) were selected based on previous literature or theoretical basis that they may be the result of multimorbidity, and also precede or cause fall-related injury (Koyanagi et al. 2014, 2021; Williams et al. 2015; Vancampfort et al. 2019; Zhao et al. 2021). Questions based on the World Mental Health Survey version of the Composite International Diagnostic Interview (Kessler and Üstün 2004) were used for the endorsement of past 12-month DSM-IV depression (American Psychiatric Association 2013). Individuals who reported to have received a lifetime diagnosis of depression and treatment for it in the past 12 months were also considered to have depression. Weak handgrip strength was defined as < 27kg for men and < 16kg for women using the average value of the two handgrip measurements of the dominant hand (Cruz-Jentoft et al. 2019). Sleep/energy, pain/discomfort, cognition, and mobility were assessed with two questions each. The actual questions can be found in supplementary Table S2. Each item was scored on a five-point scale ranging from ‘none’ to ‘extreme/cannot do’. For each separate health status, we used factor analysis with polychoric correlations to obtain a factor score which was later converted to scores ranging from 0–100 with higher values representing worse health function (Stubbs et al. 2018).

### Control variables

The control variables were selected based on past literature (Stenbacka et al. 2002; Williams et al. 2015; Ogliari et al. 2021) and included age, sex, wealth quintiles based on income, highest level of education achieved (≤ primary, secondary, ≥ tertiary), setting (rural or urban), alcohol consumption, and body mass index (BMI). Consumers of at least four (females) or five drinks (males) of any alcoholic beverage per day on at least one day in the past week were considered to be ‘heavy’ drinkers. Those who had ever consumed alcohol but were not heavy drinkers were categorized as ‘non-heavy’ drinkers (Koyanagi et al. 2015). BMI was calculated as weight in kilograms divided by height in meters squared based on measured weight and height, and classified as underweight (BMI < 18.5 kg/m^2^), normal weight (BMI 18.5–24.9 kg/m^2^), overweight (BMI 25.0–29.9 kg/m^2^), and obesity (BMI ≥ 30 kg/m^2^) in line with WHO guidelines (World Health Organization 2021b).

### Statistical analysis

The statistical analysis was done with Stata 14.2 (Stata Corp LP, College station, Texas). The analysis was restricted to those aged ≥ 50 years. The difference in sample characteristics between those with and without a fall-related injury was calculated with Chi-squared tests and Student’s *t*-tests for categorical and continuous variables, respectively. First, using the overall sample, multivariable logistic regression analyses was used to assess the association between the number of chronic conditions or individual chronic conditions (exposures) and fall-related injury (outcome). The analysis on the number of chronic conditions was also stratified by age groups (50–64 y and ≥ 65 y). Country-wise analysis was also conducted, and this used the dichotomized multimorbidity variable (i.e., ≥ 2 chronic conditions) as the exposure variable. In order to assess the degree of between-country heterogeneity in the association between multimorbidity and fall-related injury, we calculated the Higgin’s *I*^*2*^ based on country-wise estimates. This represents the degree of heterogeneity that is not explained by sampling error with values of 25%, 50%, and 75% often being considered as low, moderate, and high levels of heterogeneity (Higgins et al. 2003). Overall estimates were obtained based on country-wise estimates by meta-analysis with random effects.

Next, in order to gain an understanding of the extent to which various factors (i.e., depression, sleep/energy, pain/discomfort, cognition, mobility, and weak handgrip strength) may explain the relation between multimorbidity (i.e., ≥ 2 chronic conditions) and fall-related injury, we conducted mediation analysis, using the *khb* (Karlson Holm Breen) command in Stata (Breen et al. 2013). This method can be applied in logistic regression models and decomposes the total effect (i.e., unadjusted for the mediator) of a variable into direct (i.e., the effect of multimorbidity on fall-related injury adjusted for the mediator) and indirect effects (i.e., the mediational effect). Using this method, the percentage of the main association explained by the mediator can also be calculated (mediated percentage). Each potential mediator was included in the model individually.

All regression analyses including the mediation analysis were adjusted for age, sex, wealth, education, setting, BMI, alcohol consumption and country with the exception of the country-wise analysis which was not adjusted for country. Adjustment for country was done by including dummy variables for each country in the model as in previous SAGE publications. The model on individual chronic conditions mutually adjusted for all chronic conditions simultaneously. The sample weighting and the complex study design were taken into account in all analyses. Under 5% of the values were missing for all variables used in the study, with the exception of handgrip strength (10.1%) and BMI (6.2%). Complete-case analysis was done. Results from the regression analyses are presented as odds ratios (ORs) with 95% confidence intervals (CIs). The level of statistical significance was set at p < 0.05.

## Results

A total of 34,129 adults aged ≥ 50 years [mean (SD) age 62.4 (16.0) years; males 48.0%] were included in the current study. The sample sizes by country were: China *n* = 13,175; Ghana *n* = 4305; India *n* = 6560; Mexico *n* = 2313; Russia *n* = 3938; South Africa *n* = 3838. The sample characteristics are provided in Table 1. Overall, the prevalence of physical multimorbidity (i.e., ≥ 2 chronic physical conditions) was 47.2%, while the prevalence of fall-related injury was 4.2%. Those who had a fall-related injury were more likely to have greater number of chronic physical conditions, be older, women, have lower levels of education, and be from a rural setting. Furthermore, they were less likely to consume alcohol, more likely to be underweight, and have depression, weak handgrip strength, with worse health status in terms of sleep/energy, pain/discomfort, cognition, and mobility.

**Table 1:** Sample characteristics (overall and by fall-related injury)

Characteristic		Overall	Fall-related injury		P-value^a^
			No	Yes	
No. of chronic physical conditions	0	21.2	21.6	12.3	< 0.001
	1	31.6	32.0	23.8	
	2	22.4	22.4	22.1	
	3	12.6	12.3	18.3	
	≥ 4	12.2	11.7	23.5	
Age (years)	Mean (SD)	62.4 (16.0)	62.3 (16.0)	63.7 (14.9)	0.008
Sex	Male	48.0	48.5	35.5	< 0.001
	Female	52.0	51.5	64.5	
Wealth	Poorest	17.1	17.0	19.5	0.1532
	Poorer	19.0	18.9	20.3	
	Middle	19.5	19.4	21.3	
	Richer	21.3	21.4	20.3	
	Richest	23.1	23.3	18.6	
Education	≤ Primary	57.3	56.6	72.5	< 0.001
	Secondary	35.3	35.9	21.6	
	≥ Tertiary	7.5	7.5	5.9	
Setting	Rural	53.9	53.5	62.8	0.0013
	Urban	46.1	46.5	37.2	
Alcohol consumption	Never	67.1	66.9	72.3	0.019
	Non-heavy	28.8	29.0	24.5	
	Heavy	4.1	4.1	3.2	
Body mass index	Underweight	16.7	16.4	25.2	< 0.001
	Normal	47.6	47.7	46.3	
	Overweight	24.2	24.3	21.2	
	Obesity	11.4	11.6	7.3	
Depression	No	93.4	93.9	81.9	< 0.001
	Yes	6.6	6.1	18.1	
Weak grip strength	No	66.8	67.3	55.7	< 0.001
	Yes	33.2	32.7	44.3	
Sleep/energy^b^	Mean (SD)	27.4 (45.2)	26.9 (45.0)	39.9 (44.1)	< 0.001
Pain/discomfort^b^	Mean (SD)	30.3 (44.9)	29.6 (44.7)	45.6 (41.6)	< 0.001
Cognition^b^	Mean (SD)	30.6 (46.1)	30.1 (45.9)	42.7 (44.2)	< 0.001
Mobility^b^	Mean (SD)	32.6 (46.6)	31.9 (46.4)	47.8 (44.3)	< 0.001

SD, Standard deviation

^a^P-value was calculated by Chi-squared test and Student’s *t* tests for categorical and continuous variables, respectively

^b^Scores ranged from 0–100 with higher scores representing worse health status

**Fig. 1:**
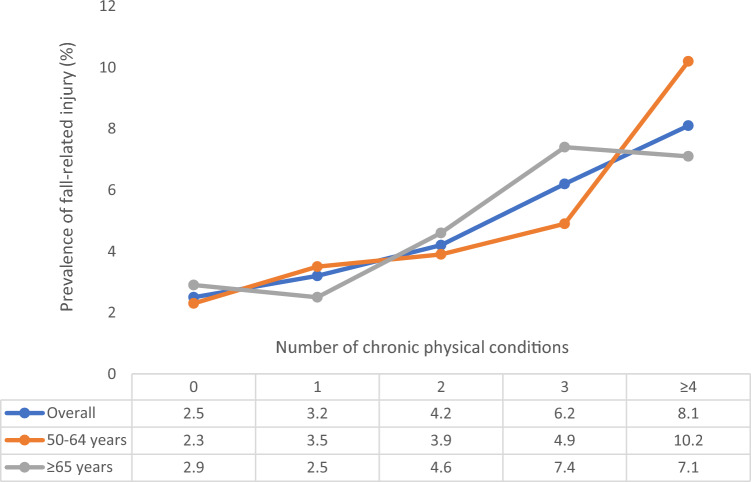
Prevalence of fall-related injury by number of chronic physical conditions (overall and by age groups)

**Fig. 2:**
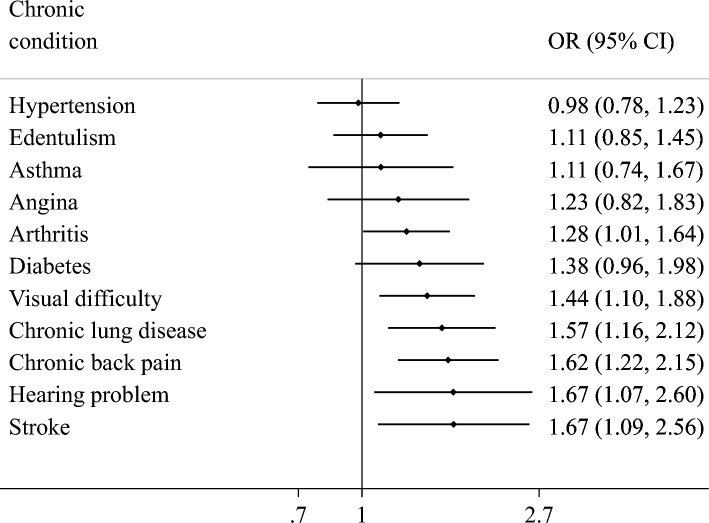
Association between individual chronic conditions and fall-related injury estimated by multivariable logistic regression. OR Odds ratio; CI Confidence interval. All the individual conditions were included simultaneously in the model. Model is adjusted for age, sex, wealth, education, setting, body mass index, alcohol consumption, and country

**Fig. 3:**
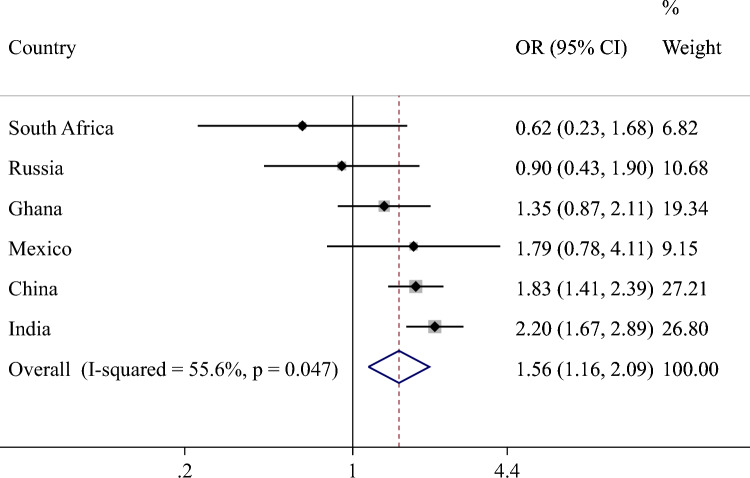
Country-wise association between multimorbidity (i.e., ≥ 2 chronic physical conditions) and fall-related injury estimated by multivariable logistic regression. OR Odds ratio; CI Confidence interval. Models are adjusted for age, sex, wealth, education, setting, body mass index, and alcohol consumption. Overall estimate was obtained by meta-analysis with random effects

Overall, the prevalence of fall-related injury increased linearly with increasing number of chronic physical conditions (Fig. 1). Specifically, the prevalence was 2.5% among those without chronic conditions but this increased to 8.1% among those with more than four chronic conditions. In terms of the individual chronic conditions, arthritis, visual difficulty, chronic lung disease, chronic back pain, hearing problems, and stroke were associated with significantly greater odds for fall-related injury (Fig. 2). Overall, compared to having no chronic conditions, having 2, 3, and ≥ 4 chronic conditions were significantly associated with 1.67 (95%CI = 1.21–2.30), 2.64 (95%CI = 1.89–3.68), and 3.67 (95%CI = 2.42–5.57) times higher odds for fall-related injury (Table 2). The association was most pronounced among those aged 50–64 years compared to older adults. Country-wise analysis showed that multimorbidity is positively associated with fall-related injury (i.e., OR > 1) in all countries with the exception of South Africa and Russia. The overall estimate based on meta-analysis was OR = 1.56 (95%CI = 1.16–2.09) with a moderate level of between-country heterogeneity. Finally, the mediation analysis showed that pain/discomfort (39.7%) explains the largest proportion of the association between multimorbidity and fall-related injury, followed by mobility (34.1%), sleep/energy (24.2%), and cognition (13.0%) (Table S3 of supplementary material). Depression (7.1%) and weak handgrip strength (2.1%) were also significant mediators but only explained the association to a lesser extent (Fig. 3).

**Table 2:** Association between number of chronic physical conditions and fall-related injury (outcome) estimated by multivariable logistic regression (overall and by age groups)

		Overall			Age 50–64 y			Age ≥ 65 y		
		OR	95%CI	P-value	OR	95%CI	P-value	OR	95%CI	P-value
No. of chronic physical conditions	0	1.00			1.00			1.00		
	1	1.34	[0.97,1.86]	0.075	1.61	[1.07,2.43]	0.022	0.82	[0.45,1.50]	0.524
	2	1.67	[1.21,2.30]	0.002	1.71	[1.13,2.59]	0.011	1.46	[0.86,2.50]	0.163
	3	2.64	[1.89,3.68]	< 0.001	2.28	[1.41,3.67]	0.001	2.59	[1.50,4.46]	0.001
	≥ 4	3.67	[2.42,5.57]	< 0.001	4.35	[2.50,7.58]	< 0.001	2.90	[1.66,5.08]	< 0.001

OR, Odds ratio; CI, Confidence interval

Models are adjusted for age, sex, wealth, education, setting, body mass index, alcohol consumption, and country

## Discussion

### Main findings

In this large sample of older adults from six LMICs, it was found that arthritis, visual difficulty, chronic lung disease, chronic back pain, hearing problems, and stroke were associated with significantly greater odds for fall-related injury. Moreover, in the overall sample, those having 2, 3, and ≥ 4 chronic conditions were significantly more likely to experience a fall-related injury. Country-wise analysis showed that there is a moderate level of between-country heterogeneity in the association between multimorbidity (i.e., ≥ 2 chronic physical conditions) and fall-related injury. This association was mainly explained by pain/discomfort (mediated% 39.7%), mobility (34.1%), sleep/energy (24.2%), cognition (13.0%), while depression (7.1%) and weak handgrip strength (2.1%) explained it to a lesser extent.

### Interpretation of the findings

Findings from the present study both support and add to previous literature. They support previous literature through confirming that an association exists between physical multimorbidity and falls (Shumway-Cook et al. 2009; Sibley et al. 2014; Clerencia-Sierra et al. 2015; Teixeira et al. 2019; Immonen et al. 2020; Barik et al. 2022) and add to this by showing that such an association exists across several different contexts, namely in a large multi-country sample of older adults from low-and middle-income regions.

Several plausible pathways exist for the associations between individual chronic conditions and fall-related injury observed in our study. First, arthritis is likely associated with injurious falls owing to pain and musculoskeletal symptoms that lead to decreased use of the affected limbs and joints, muscle weakness, postural imbalance, and poor functional performance, ultimately leading to an increased risk of falls (Doré et al. 2015). Second, visual difficulties likely lead to an increased risk of injurious falls owing to the impact of sight impairment on tripping/stumbling while getting around the home and navigating the environment away from home (Brundle et al. 2015). Third, chronic lung disease may increase risk of fall-related injury owing to lower limb muscle weakness and impaired activities of daily living as well as intrinsic risk factors such as gait and balance deficits, nutritional depletion, malnutrition, depression, cognitive impairments and medications (Roig et al. 2009). When considering chronic back pain, a higher risk of fall-related injury may be owing to static or gait imbalances (Liu-Ambrose et al. 2002). In terms of hearing problems, concomitant dysfunction of both the cochlear and vestibular sense organs, given their shared location within the bony labyrinth of the inner ear, may increase risk of falls. Furthermore, decreased hearing sensitivity may also directly limit access to auditory cues that are needed for environmental awareness (Lin and Ferrucci 2012). Finally, the association between stroke and fall-related injury may be explained by impaired mobility (Wei et al. 2019).

The present study found that multimorbidity increased the risk of injurious falls in a linear fashion. This suggests an additive effect of chronic disease on fall risk, irrespective of the specific condition. Multimorbidity likely contributes to increased risk of injurious falls owing to similar pathways for individual chronic conditions. Greater number of chronic conditions likely results in a higher number of potential pathways that can lead to falls, and this may increase risk of falls linearly. Specifically, in our study, we identified several potential mediators of the multimorbidity-injurious falls relationship, with pain/discomfort, mobility, sleep/energy, and cognition explaining a large proportion of the association. Multimorbidity is indeed likely to increase risk of pain/discomfort in older adults via symptom burden (Nakad et al. 2020), and pain/discomfort can increase risk of falling. Specifically, pain contributes to functional decline and muscle weakness, and is associated with mobility limitations that could predispose a person to falling (Leveille et al. 2009). Multimorbidity can result in mobility limitations by accelerating the decline in functional ability (Wang et al. 2021). Importantly, low levels of functional ability are associated with a higher risk of falls owing to factors such as gait impairments (McGough et al. 2013). Sleep/energy complications can result from multimorbidity as older adults are particularly susceptible to insomnia given the change in sleep architecture associated with age. Specifically, lighter sleep renders them increasingly vulnerable to sleep disruption caused by physical chronic conditions (McCrae and Lichstein 2001). Insufficient sleep, in turn, is associated with increased risk of injurious falls due to impaired cognitive function, depression, balance problems, and use of medications (Stone et al. 2014). Next, cognitive decline is often observed among those with multimorbidity and this can be explained via several pathways; one being vascular effects on frontal lobe functions (Fabbri et al. 2016). In turn, cognitive decline can increase risk of fall-related injury; for example, gait requires executive functioning and attention, and deteriorates during dual tasking (Wei et al. 2020). Finally, other factors which were not assessed in our study but may be important in the multimorbidity-injurious fall association include factors such as polypharmacy (Hammond and Wilson 2013) and frailty (Hanlon et al. 2018). Importantly, many common medications can increase fall-risk particularly those prescribed for sleep and pain relief (Harvard Health Publishing 2021). Frailty can increase fall-risk via a loss of muscle strength and power (Clegg et al. 2013).

Interestingly, we observed a moderate level of between-country heterogeneity in the association between multimorbidity and fall-related injury. Although the reason for this is unclear, it may be related to different disease profiles between countries or differences in availability of treatment. For example, a previous study using the SAGE dataset found that multimorbidity patterns differ substantially by country (Garin et al. 2016). While we can only speculate, it is possible that the null association between multimorbidity and injury-related falls in South Africa and Russia may be related to the fact that these countries had higher prevalence of hypertension, which in our study was the chronic condition least associated with falls (Garin et al. 2016).

### Public health and clinical implications

Findings from the present study suggest that those with multimorbidity should be targeted with fall reduction interventions. The present findings also suggest that prevention interventions to reduce fall-related injury in this population should focus on pain reduction, improving mobility, improving sleep/energy and aiding in the prevention of accelerated cognitive decline. When considering such conditions, effective interventions may wish to utilize strength-based exercise. Indeed, strength-based exercise among older adults has been shown to be effective in improving all of the mentioned target variables (Nelson et al. 2004; Jansen et al. 2011; Chupel et al. 2017; Kovacevic et al. 2018). In addition to such a lifestyle intervention, clinicians and geriatricians should be aware that those with multimorbidity are at a significant increased risk of fall-related injury and should consider this when prescribing.

### Strengths and limitations

The large sample of older adults from six LMICs is a clear strength of the present study, as well as the identification of important mediators in the multimorbidity/injurious falls relationship. However, findings from this study should be interpreted in light of the studies limitations. First, there was limited data on some chronic conditions so some important conditions such as cancer could not be taken into consideration. Second, the study was cross-sectional in nature so caution should be taken when interpreting the findings and causation cannot be assumed. Next, some level of bias could have been introduced due to missing values. In particular, the percentage of those who were missing values on handgrip strength and BMI were higher than other variables. This may imply that those missing values on these variables include a higher proportion of people who could not participate in objective health assessments due to illness. Finally, variables were self-reported and are therefore susceptible to recall and social desirability bias.

## Conclusion

In this large sample of older adults from six LMICs, it was found that arthritis, visual difficulty, chronic lung disease, chronic back pain, hearing problems, and stroke were associated with significantly greater odds for fall-related injury. Moreover, those with multimorbidity were significantly more likely to experience a fall-related injury. A large proportion of this association was explained by pain/discomfort, mobility, sleep/energy, and cognition. In older adults with multimorbidity, interventions to address these conditions such as strength training interventions may be effective in reducing fall-related injury. Clinicians should be aware those with multimorbidity are at an increased risk of falls, especially when prescribing medication.

## Supplementary Information

Below is the link to the electronic supplementary material.

Supplementary file1

## Data Availability

The data that support the findings of this study are available from the corresponding author upon reasonable request.
